# Visual attention during post-handover bedside observation of critically ill patients: an eye-tracking comparison across emergency and critical care nursing experience groups

**DOI:** 10.1186/s12912-026-05324-1

**Published:** 2026-09-02

**Authors:** Takafumi Noguchi, Hidekazu Hishinuma, Jin Kikuchi, Ryo Kayashima, Tetsuya Nakada, Tomomi Muraoka, Sadami Momiyama, Michiko Saito, Toshimi Sairenchi, Koji Wake

**Affiliations:** 1 Department of Adult Nursing, Dokkyo Medical University School of Nursing, 880 Kitakobayashi, Mibu, Shimotsuga-gun, Tochigi, 321-0293 Japan; 2 Department of Public Health, Dokkyo Medical University School of Medicine, Tochigi, Japan; 3 Department of Emergency and Critical Care Medicine, Dokkyo Medical University School of Medicine, 880 Kitakobayashi, Mibu, Shimotsuga-gun, Tochigi 321-0293 Japan; 4 Department of Fundamental Nursing, Dokkyo Medical University School of Nursing, Tochigi, Japan; 5 Nursing Department, Dokkyo Medical University Hospital, Tochigi, Japan; 6 Department of Fundamental Clinical Nursing, Dokkyo Medical University School of Nursing, Tochigi, Japan; 7 Medical Science of Nursing, Dokkyo Medical University School of Nursing, Tochigi, Japan

**Keywords:** Critical care nursing, Eye tracking, Gaze behavior, Patient observation, Post-handover bedside observation, Visual attention

## Abstract

**Background:**

Post-handover bedside observation is routine in critical care, but experience-related differences in gaze behavior during this observation remain unclear. This study compared gaze behavior according to emergency and critical care nursing experience.

**Methods:**

This cross-sectional observational study was conducted in a Japanese emergency and critical care center between September 2022 and February 2024. Sixty-six nurses were classified into four experience groups: <1 year, 1–<4 years, 4–<9 years, and ≥ 9 years. Eighteen mechanically ventilated patients contributed 66 observation sessions. Mobile eye tracking measured fixation duration, fixation count, and visit count across 13 areas of interest. Fixation duration was expressed as a percentage of observation time, while count outcomes were analyzed using negative binomial generalized estimating equations with observation duration as an offset and patient-level clustering. The Benjamini–Hochberg procedure controlled the false discovery rate across 42 tests.

**Results:**

After false discovery rate correction, significant differences among experience groups remained for five fixation-duration, four fixation-rate, and six visit-rate measures. Pairwise differences in fixation duration and fixation rate varied in direction across areas of interest. In contrast, all significant visit-rate comparisons involving the < 1-year group showed lower rates, including for the lower limbs, insertion sites, physiological measurement devices, infusion lines and pumps, and urinary catheter. Total visit rate was also lower in the < 1-year group than in the 4–<9-year group. This categorical association remained significant after covariate adjustment and in a generalized linear mixed-model sensitivity analysis. When experience was modeled numerically with covariate adjustment, the association was attenuated (*p* = 0.055).

**Conclusions:**

Experience-related differences were observed across gaze measures, with visit rate demonstrating the most directionally consistent pattern. Nurses with < 1 year of experience showed fewer gaze entries per unit time into several patient-, device-, and treatment-related areas than nurses in a more-experienced group. The findings suggest that experience-related differences may involve not only the amount of visual attention directed to individual areas but also the frequency with which nurses direct gaze toward multiple potential sources of patient information. Gaze behavior should not be interpreted as a direct measure of clinical competence, observation quality, or clinical judgment.

**Supplementary Information:**

The online version contains supplementary material available at https://doi.org/10.1186/s12912-026-05324-1.

## Background

In critical care settings, nurses care for patients with severe and rapidly changing conditions who often require close monitoring and complex interventions [1]. Patient observation is therefore central to acute and critical care nursing, supporting patient safety, early recognition of deterioration, and clinical judgment [2, 3]. Previous studies have described patient observation in critical care as involving multiple skills and have examined patient observation skills and knowledge as components of critical care nursing competence [4–6]. In intensive care units (ICUs), recognizing subtle or patient-specific changes is essential for preventing further deterioration [7]. Professional standards for acute and critical care nursing also identify assessment, data collection, and ongoing evaluation of patient status as core nursing responsibilities [8].

Bedside clinical handover and shift-to-shift handoffs support the transfer of information and responsibility, continuity of care, and patient safety [9, 10]. In emergency nursing, effective handover has been described as individualized, structured, conducted at the bedside, and inclusive of treatment information, nursing observations, and care plans [11]. After handover or at the start of patient assignment, critical care nurses commonly perform a routine bedside observation to grasp the patient’s overall condition and treatment-monitoring environment. In the present study, this practice is referred to as post-handover bedside observation. This observation may provide an early opportunity to visually encounter patient- and treatment-related information that may subsequently support cue recognition and interpretation. Tanner’s clinical judgment model describes noticing as an early component of clinical judgment that is influenced by nurses’ previous experience, knowledge of the patient, expectations, and clinical context [12]. The National Council of State Boards of Nursing Clinical Judgment Measurement Model (NCJMM) similarly identifies recognizing cues and analyzing cues as early cognitive processes in clinical judgment [13, 14].

Despite being a familiar part of nursing practice, post-handover bedside observation is rarely described in detail. During such observation, nurses may need to inspect the patient’s body, invasive devices, monitoring equipment, ventilation-related information, infusion systems, and treatment-related prescriptions within a limited period. Unlike tasks with a relevant pre-specified target or abnormality, this observation is open-ended: nurses must determine which information sources warrant attention in the context of the individual patient. Because post-handover bedside observation is embedded in routine clinical workflow, it may be shaped by nurses’ experience, prior knowledge, and clinical priorities. Differences in how nurses pay attention to these multiple bedside information sources are difficult to capture through conventional observation or self-report.

Eye-tracking technology offers a way to quantify gaze behavior during clinical tasks. Fixations and visits can be used to describe how visual attention is allocated across areas of interest (AOIs) [15, 16]. In nursing research, eye tracking has been applied in clinical education, simulation, and performance evaluation [17]. In critical care, non-simulated studies have described the distribution of nurses’ visual attention during routine ICU care, whereas experience-related differences have been examined during a constrained closed-loop ventilation task [18, 19]. Handover-related eye-tracking studies have also examined simulated shift-start safety checks in which predefined errors had to be identified [20]; a recent randomized simulation study examined the effects of time pressure on error detection and gaze behavior [21].

These tasks differ in nature from naturally occurring post-handover bedside observation, in which nurses observe actual patients without predefined abnormalities, a prescribed observation sequence, or a fixed observation duration. Consequently, it remains unclear whether nursing experience is associated with differences in how visual attention is directed among multiple patient-, device-, monitoring-, and treatment-related areas during this open-ended clinical observation. Therefore, this study aimed to compare gaze behavior during post-handover bedside observation according to emergency and critical care nursing experience. Specifically, we used mobile eye tracking to examine fixation duration, fixation count, and visit count across AOIs during post-handover bedside observation of critically ill patients.

## Methods

### Study design and setting

This cross-sectional observational study used mobile eye tracking to examine gaze behavior during post-handover bedside observation among critical care nurses. Reporting was guided by the Strengthening the Reporting of Observational Studies in Epidemiology (STROBE) statement [22].

Data were collected between September 2022 and February 2024 in the intensive care area of the Emergency and Critical Care Center at Dokkyo Medical University Hospital, a tertiary hospital in Japan. The unit provides care for critically ill medical and surgical patients. Recordings were made during routine clinical practice, and no task sequence, observation order, or observation duration was specified for the study.

### Participants and patients

Participants were full-time nurses working in the emergency and critical care center who agreed to participate and were able to perform routine clinical work while wearing mobile eye-tracking glasses. Nurses were excluded if they had factors likely to interfere with eye tracking, such as colored contact lenses, false eyelashes, or other eye-area characteristics that could affect gaze recording. Written informed consent was obtained from all participating nurses before data collection. Of the 67 nurses who agreed to participate, one was excluded from the analysis because the gaze data required for analysis were not obtained; thus, 66 nurses were included in the final analysis.

Patients were eligible if they were intubated and receiving mechanical ventilation, and if their clinical condition was judged by the attending physician, certified critical care nurses, and nurse managers to be stable enough for the study procedure. Written informed consent was obtained from each patient or a family member acting as a surrogate decision-maker. In this study, multiple nurses could observe the same patient.

Participants reported their current year of emergency and critical care nursing experience. Each nurse contributed one observation session. Experience was operationally categorized into four groups corresponding to the first year, second–fourth year, fifth–ninth year, and tenth year or later: <1 year, 1–<4 years, 4–<9 years, and ≥ 9 years of elapsed experience, respectively. The grouping was intended to distinguish the initial, early, intermediate, and longer periods of emergency and critical care nursing experience. This was based on Benner’s model of skill acquisition and previous critical care nursing research showing experience-related differences in patient observation skills [5, 23], but the cut-points were not intended to correspond directly to validated levels of competence or expertise. The study protocol specified a target sample size of 16 participants per group based on a calculation using G*Power 3 [24].

### Post-handover bedside observation

We defined post-handover bedside observation as the initial bedside observation performed by a nurse after handover or at the start of patient assignment to grasp the patient’s overall condition and treatment-monitoring environment. Distinct from scheduled vital sign measurement or a formal comprehensive assessment, post-handover bedside observation included observation of consciousness, respiratory and circulatory status, physical findings, invasive devices, the mechanical ventilator, monitoring devices, infusion lines and pumps, urinary catheter, and treatment-related prescriptions.

Measurements were performed when a participating nurse conducted post-handover bedside observation of an eligible patient after handover or at the start of patient assignment as part of routine practice. After wearing the eye-tracking device and completing calibration, the nurse moved to the bedside and conducted the observation according to the usual clinical workflow. To preserve the natural flow of routine care, the research team did not instruct nurses on what to observe, in what order, or for how long. To reduce behavioral reactivity, participants were not instructed to look at specific bedside areas, and the AOIs used for analysis were not explained to participants before recording.

**Fig. 1:**
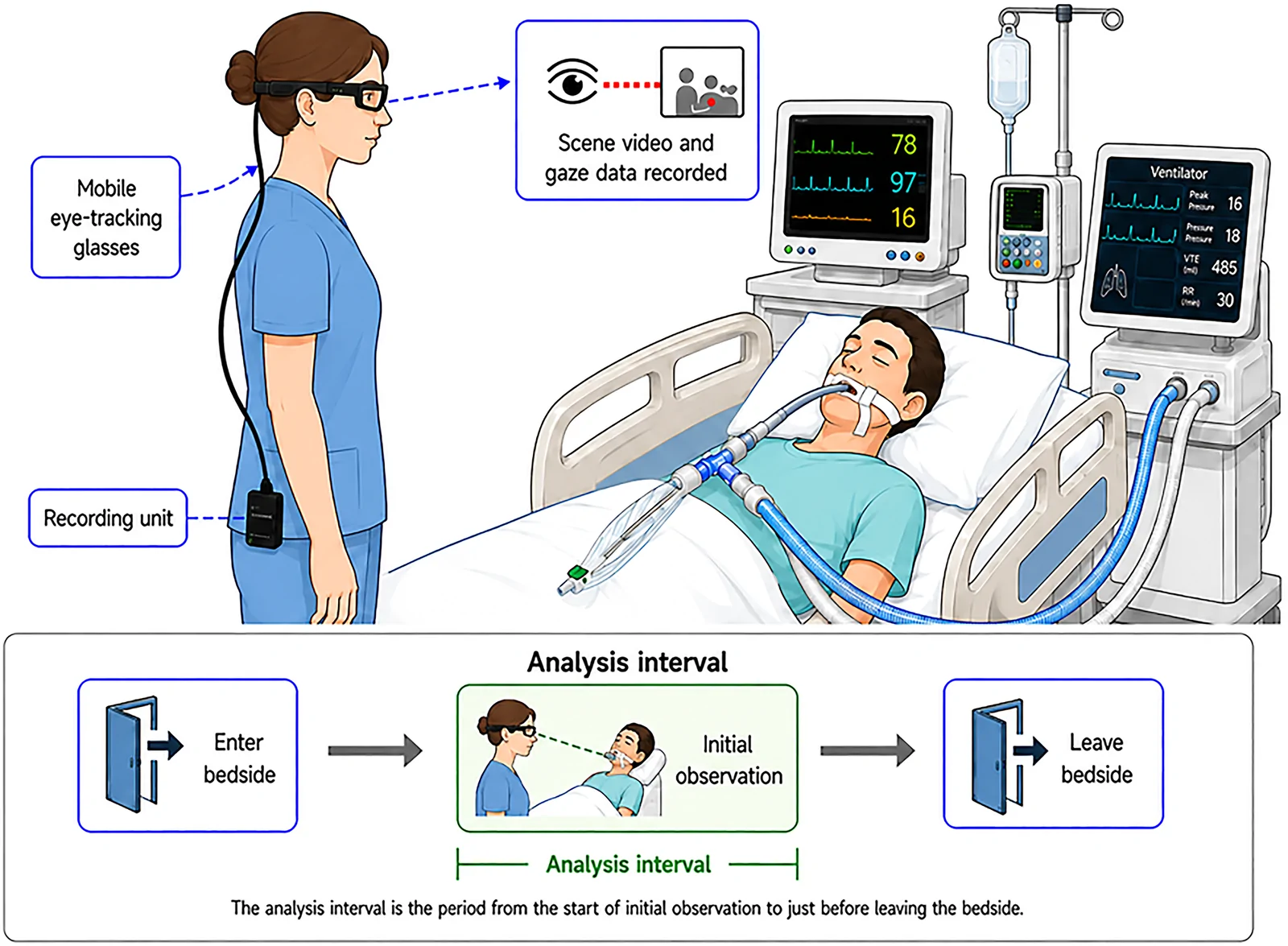
Eye-tracking setup and analysis interval for post-handover bedside observation. The nurses wore mobile eye-tracking glasses during routine bedside observation after handover or at the start of patient assignment. Scene video and gaze data were recorded using a mobile eye-tracking system. The analysis interval was defined as the period from entering the bedside area to the completion of post-handover bedside observation

The analysis interval extended from entry into the bedside area to completion of post-handover bedside observation. Completion was determined retrospectively from the scene video and gaze recording, using the final gaze within any predefined bedside AOI as an anchor and the subsequent scene context to confirm that the observation episode had ended, rather than that gaze had only temporarily shifted away. Completion typically coincided with the nurse leaving the bedside or transitioning to another activity. Activities outside direct bedside observation, such as documentation in the electronic medical record, information checking away from the patient, or work-related communication outside bedside observation, were excluded from analysis. Figure 1 illustrates the eye-tracking setup and analysis interval.

### Eye-tracking procedure

Gaze behavior was recorded using Tobii Pro Glasses 3 (Tobii AB, Sweden), a wearable eye-tracking device with a sampling rate of 50 Hz and an accuracy of ± 0.6° [25]. The system consists of eye-tracking glasses and a waist-mounted recording unit. Data were collected using the Controller application (version 1.16.4) according to the manufacturer’s instructions [25].

Before each recording, calibration was performed by asking the participant to fixate on a target placed approximately 50–100 cm away. Calibration was considered successful when the indicator in the Controller application changed from red to green. If calibration was insufficient, the position of the device was adjusted and calibration was repeated.

Scene videos were recorded in 1080p and stored with gaze coordinate data. After recording, the data were imported into Tobii Pro Lab (version 24.21) for processing and analysis. Gaze events were identified using the Velocity-Threshold Identification (I-VT) filter in Tobii Pro Lab. Fixations were treated as periods in which gaze was relatively stable [15, 16]. In this study, fixation events were extracted using a minimum duration of 60 ms.

### Data cleaning

Gaze data were initially screened and processed by the primary analyst, who mapped them to the predefined AOIs and identified the analysis-interval boundaries. All 66 included recordings were subsequently reviewed by a three-member analysis team, including the primary analyst, to confirm the interval boundaries and gaze-to-AOI mapping. Ambiguous or discrepant judgments were resolved through discussion and consensus.

### Areas of interest and gaze metrics

Three researchers first reviewed recordings from five cases and identified candidate visual targets during post-handover bedside observation. Provisional AOIs were established through discussion among the three researchers. The provisional AOIs were then applied to all recordings, after which the three researchers reviewed the full data set to confirm their appropriateness and finalized the AOI definitions by consensus. The AOIs were defined for analytic purposes and were not used to instruct participants before or during bedside observation.

**Fig. 2:**
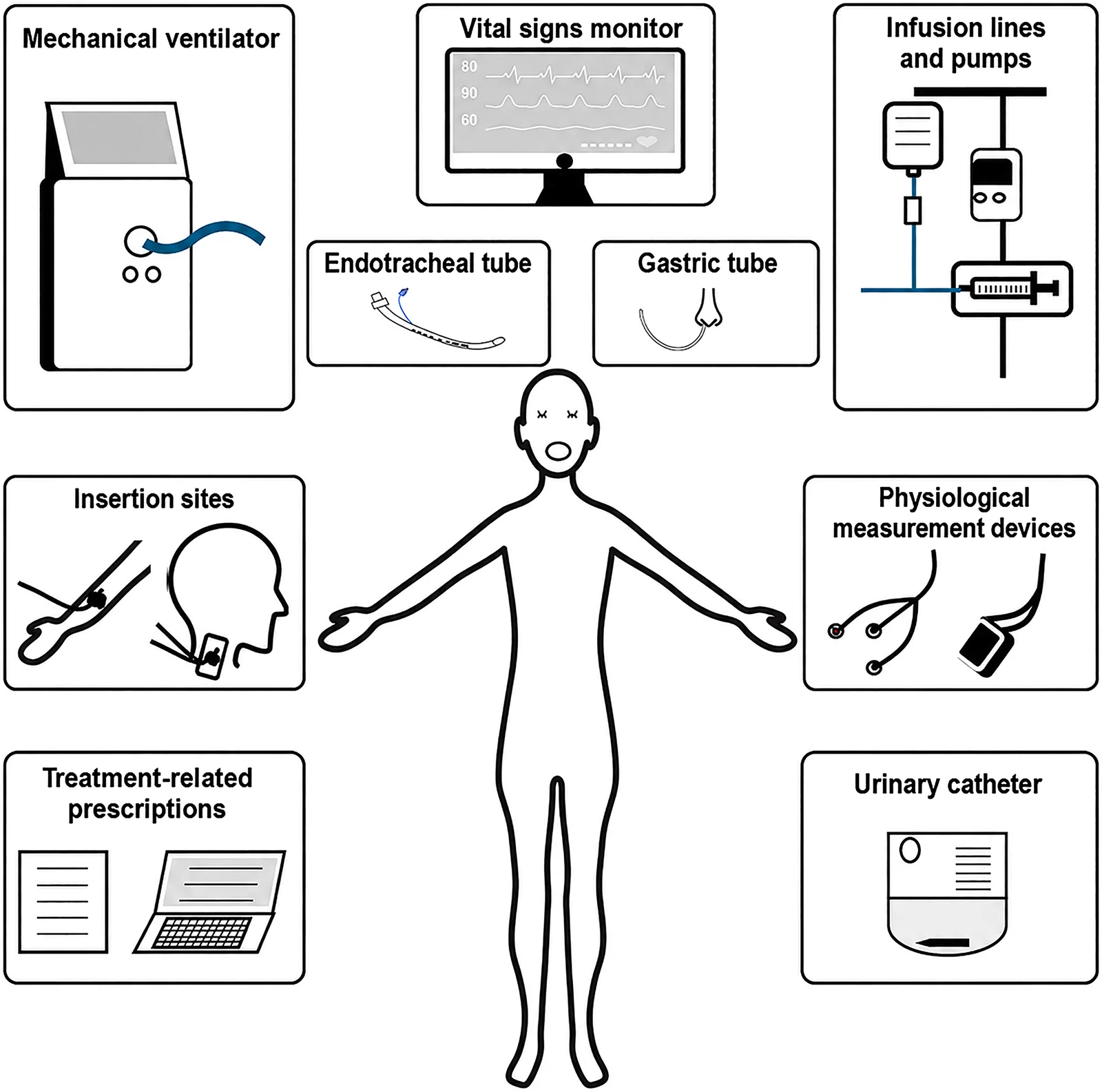
Areas of interest used for gaze analysis during post-handover bedside observation. The figure shows the areas of interest (AOIs) used to classify nurses’ gaze behavior during post-handover bedside observation. AOIs included patient body regions and bedside equipment or information sources, such as insertion sites, the endotracheal tube, gastric tube, vital signs monitor, mechanical ventilator, physiological measurement devices, infusion lines and pumps, urinary catheter, and treatment-related prescriptions

Thirteen AOIs were defined: face, upper limbs, lower limbs, chest/abdomen, insertion sites, endotracheal tube, gastric tube, vital signs monitor, mechanical ventilator, physiological measurement devices, infusion lines and pumps, urinary catheter, and treatment-related prescriptions. The face AOI included facial expression and skin color. The upper and lower limb AOIs included limb condition and movement. The chest/abdomen AOI included respiratory and abdominal findings. Insertion sites included peripheral intravenous lines, central venous lines, arterial lines, and related insertion areas. The vital signs monitor AOI included monitor values and waveforms. The mechanical ventilator AOI included ventilator settings and alarms. Physiological measurement devices included equipment and accessories used to measure physiological parameters, such as noninvasive blood pressure cuffs, electrocardiogram electrodes and leads, pulse oximetry sensors, and arterial pressure monitoring components. Infusion lines and pumps included infusion devices, infusion lines, and flow-related equipment. Treatment-related prescriptions included physician orders, infusion prescriptions, and ventilator-related prescriptions. All 13 AOIs were physically present and observable in every included observation session. Accordingly, zero values for fixation- or visit-based metrics indicated that no corresponding gaze event was recorded within an available AOI, rather than that the AOI was absent or unobservable. Figure 2 illustrates the AOIs used for gaze analysis during post-handover bedside observation.

The gaze metrics were fixation duration, fixation count, and visit count for each AOI. Fixation duration was defined as the total duration of fixations within an AOI. Fixation count was defined as the number of fixation events detected within an AOI. Visit count was defined as the number of times gaze entered an AOI; a visit began when gaze entered the AOI and ended when gaze left the AOI after one or more fixations [15]. Analysis interval duration was calculated for each observation session. For descriptive presentation, fixation duration was expressed as a percentage of the analysis interval, and fixation count and visit count were expressed as rates per minute. Total values across the 13 AOIs were also calculated for each gaze metric.

### Statistical analysis

Nurse characteristics were summarized using means and standard deviations or medians and interquartile ranges for continuous variables, as appropriate, and frequencies and percentages for categorical variables. Differences among experience groups were examined using the Kruskal–Wallis test for continuous variables and Fisher’s exact test for categorical variables. Patient characteristics were summarized descriptively at the observation-session level. Because the same patient could contribute to observations involving nurses from different experience groups, inferential between-group comparisons were not performed for patient characteristics.

Analysis interval duration and time-standardized gaze metrics were summarized by experience group. Fixation duration was presented as the percentage of the analysis interval, whereas fixation and visit counts were presented as rates per minute.

Primary analyses were conducted for all 13 AOIs and the total measure for each of the three gaze metrics. Because multiple nurses could observe the same patient, generalized estimating equations (GEE) were used with patient ID specified as the clustering variable and an exchangeable working correlation structure with robust covariance estimation. For fixation duration, the percentage of the analysis interval was modeled using a normal distribution with an identity link. Fixation count and visit count were modeled as count outcomes using a negative binomial distribution with a log link; the natural logarithm of analysis interval duration in minutes was included as an offset to account for differences in observation duration. Accordingly, model estimates for these count outcomes were interpreted as fixation rates and visit rates per minute.

The primary analyses comprised 42 omnibus tests: 13 AOIs plus the total measure for each of the three gaze metrics. The Benjamini–Hochberg procedure was applied across all 42 omnibus tests to control the false discovery rate (FDR) [26]. An FDR-adjusted *q < 0.05* was considered statistically significant. For outcomes that remained significant after FDR correction, pairwise comparisons between experience groups were performed with Bonferroni adjustment. Sensitivity analyses focused on total visit rate, the central outcome showing the most directionally consistent group pattern. The categorical experience-group model was additionally adjusted for patient age, patient sex, and ICU length-of-stay category; a negative binomial generalized linear mixed model (GLMM) with a patient-level random intercept and analysis-duration offset was also fitted. Finally, emergency and critical care nursing experience was modeled as a numeric predictor using participants’ self-reported current year of experience, without and with adjustment for patient age, patient sex, and ICU length of stay.

All statistical analyses, including the Benjamini–Hochberg calculations, were performed using IBM SPSS Statistics for Windows, version 28.0 (IBM Corp., Armonk, NY, USA). All tests were two-sided. Statistical significance was defined as *p* < 0.05 for analyses not subject to FDR correction and *q* < 0.05 for the 42 primary omnibus tests.

### Use of generative AI tools

ChatGPT (OpenAI, San Francisco, CA, USA) was used only to support the preparation of a preliminary draft of Fig. 1. DeepL Write was used to support English-language editing and proofreading. All AI-assisted outputs were reviewed, verified, and revised by the authors, who take full responsibility for the manuscript.

## Results

### Participant and patient characteristics

A total of 67 nurses agreed to participate. One participant was excluded because gaze data required for analysis were not obtained. The final analysis included 66 nurses and 66 observation sessions involving 18 mechanically ventilated patients. Each nurse contributed one observation session. Nurses were classified into four groups according to emergency and critical care nursing experience: <1 year (*n* = 23), 1–<4 years (*n* = 15), 4–<9 years (*n* = 16), and ≥ 9 years (*n* = 12). Nurse and patient characteristics are summarized in Table 1.

**Table 1:** Nurse and patient characteristics by emergency and critical care nursing experience group

Characteristic	Total (*n* = 66)	< 1 year (*n* = 23)	1–<4 years (*n* = 15)	4–<9 years (*n* = 16)	≥ 9 years (*n* = 12)	*p* value
**Panel A. Nurse characteristics**						
**Sex, ** ***n*** ** (%)**						
Female	49 (74.2)	16 (69.6)	14 (93.3)	12 (75.0)	7 (58.3)	0.19
Age, years, mean ± SD	29.5 ± 7.3	25.4 ± 5.2	26.5 ± 4.2	26.7 ± 3.9	41.0 ± 5.0	< 0.01
**Dominant hand**,** n (%)**						
Right hand	61 (92.4)	21 (91.3)	15 (100.0)	15 (93.8)	10 (83.3)	0.44
Left hand	5 (7.6)	2 (8.7)	0 (0.0)	1 (6.2)	2 (16.7)	
**Number of times caring for the observed patient**,** n (%)**						
1	59 (89.4)	20 (87.0)	14 (93.3)	15 (93.8)	10 (83.3)	0.09
2	5 (7.6)	3 (13.0)	1 (6.7)	1 (6.2)	0 (0.0)	
3	2 (3.0)	0 (0.0)	0 (0.0)	0 (0.0)	2 (16.7)	
**Panel B. Patient characteristics**						
**Sex**,** n (%)**						
Female	30 (45.5)	5 (21.7)	6 (40.0)	8 (50.0)	11 (91.7)	
Age, years, mean ± SD	77.8 ± 8.7	76.1 ± 6.6	78.8 ± 7.6	74.5 ± 11.5	84.0 ± 6.4	
**Length of ICU stay**,** days**,** n (%)**						
1–4	16 (24.2)	3 (13.0)	1 (6.7)	8 (50.0)	4 (33.3)	
5–9	29 (43.9)	9 (39.1)	11 (73.3)	3 (18.8)	6 (50.0)	
10–14	12 (18.2)	7 (30.4)	1 (6.7)	3 (18.8)	1 (8.3)	
15–19	5 (7.6)	1 (4.3)	1 (6.7)	2 (12.5)	1 (8.3)	
≥20	4 (6.1)	3 (13.0)	1 (6.7)	0 (0.0)	0 (0.0)	
**Disease category**,** n (%)**						
Cardiovascular diseases	17 (25.8)	8 (34.8)	4 (26.7)	4 (25.0)	1 (8.3)	
Trauma	13 (19.7)	3 (13.0)	3 (20.0)	4 (25.0)	3 (25.0)	
Respiratory diseases	29 (43.9)	8 (34.8)	6 (40.0)	7 (43.8)	8 (66.7)	
Digestive diseases	2 (3.0)	1 (4.3)	1 (6.7)	0 (0.0)	0 (0.0)	
Nervous system diseases	5 (7.6)	3 (13.0)	1 (6.7)	1 (6.2)	0 (0.0)	
**Medical devices and equipment**,** n (%)**						
Ventilator	66 (100.0)	23 (100.0)	15 (100.0)	16 (100.0)	12 (100.0)	
Infusion pump	66 (100.0)	23 (100.0)	15 (100.0)	16 (100.0)	12 (100.0)	
Syringe pump	62 (93.9)	20 (87.0)	15 (100.0)	15 (93.8)	12 (100.0)	
Electric low-pressure suction device	3 (4.5)	1 (4.3)	1 (6.7)	1 (6.2)	0 (0.0)	
Endotracheal tube	66 (100.0)	23 (100.0)	15 (100.0)	16 (100.0)	12 (100.0)	
Gastric tube	66 (100.0)	23 (100.0)	15 (100.0)	16 (100.0)	12 (100.0)	
Arterial line	66 (100.0)	23 (100.0)	15 (100.0)	16 (100.0)	12 (100.0)	
Central venous line	19 (28.8)	5 (21.7)	5 (33.3)	4 (25.0)	5 (41.7)	
**Medications**,** n (%)**						
Antihypertensive	26 (39.4)	12 (52.2)	9 (60.0)	2 (12.5)	3 (25.0)	
Vasopressors	15 (22.7)	2 (8.7)	2 (13.3)	8 (50.0)	3 (25.0)	
Muscle relaxants	8 (12.1)	2 (8.7)	2 (13.3)	4 (25.0)	0 (0.0)	
Sedatives/analgesics	38 (57.6)	12 (52.2)	9 (60.0)	9 (56.2)	8 (66.7)	

Values are presented as mean ± SD or *n* (%), as appropriate. For nurse characteristics, *p* values were calculated using the Kruskal–Wallis test for continuous variables and Fisher’s exact test for categorical variables. Patient characteristics are presented descriptively at the observation-session level because 18 unique patients contributed to 66 observation sessions and individual patients could contribute to more than one nursing experience group; inferential between-group tests were therefore not performed for patient characteristics. Experience-group labels correspond to the first year, second–fourth year, fifth–ninth year, and tenth year or later, respectively. ICU, intensive care unit; SD, standard deviation

Because individual patients could contribute to more than one observation session and could be observed by nurses from different experience groups, patient characteristics were presented descriptively without inferential between-group comparisons.

### Observation duration and descriptive gaze measures

The mean analysis interval was 8.29 ± 3.56 min, with a median of 8.51 min and a range of 1.37–18.75 min. Descriptive statistics for fixation duration, fixation rate, and visit rate standardized for the analysis interval are shown in Table 2.

**Table 2:** Time-standardized gaze metrics during post-handover bedside observation by nursing experience group

Gaze metric / AOI	< 1 year (*n* = 23)	1–<4 years (*n* = 15)	4–<9 years (*n* = 16)	≥ 9 years (*n* = 12)
**Analysis interval duration**,** min**	8.47 (4.71–9.63)	7.16 (6.04–10.53)	8.66 (6.15–11.40)	8.18 (6.28–9.68)
**Fixation duration**,** % of analysis interval**				
Face	5.30 (3.33–8.13)	6.65 (4.18–8.01)	7.74 (5.26–10.92)	5.37 (2.77–6.91)
Upper limbs	4.41 (0.97–6.39)	1.91 (1.41–3.17)	3.63 (2.31–8.93)	3.60 (2.70–4.32)
Lower limbs	1.40 (0.18–3.71)	2.44 (0.85–3.86)	2.91 (1.13–3.69)	1.64 (1.27–3.79)
Chest/abdomen	5.91 (2.50–11.40)	8.06 (4.79–12.07)	10.59 (6.38–12.00)	9.16 (4.30–10.01)
Insertion sites	1.02 (0.00–1.92)	2.90 (1.83–3.16)	2.14 (1.08–2.68)	2.55 (0.86–2.83)
Endotracheal tube	3.80 (1.45–5.03)	2.57 (1.38–2.99)	3.06 (2.64–3.82)	2.62 (1.63–3.28)
Gastric tube	2.51 (1.62–3.53)	1.87 (1.12–2.70)	1.49 (0.63–2.08)	0.98 (0.52–2.66)
Vital signs monitor	4.44 (1.99–6.50)	4.44 (2.89–7.66)	5.18 (3.50–7.29)	4.94 (3.79–7.78)
Mechanical ventilator	2.08 (1.57–9.90)	3.98 (1.74–7.15)	4.30 (2.31–6.69)	4.94 (2.58–6.48)
Physiological measurement devices	1.09 (0.10–2.66)	2.14 (0.64–3.33)	1.81 (0.87–3.01)	2.77 (1.09–5.32)
Infusion lines and pumps	8.58 (5.10–15.82)	12.24 (9.16–20.65)	11.47 (6.75–17.71)	14.86 (11.55–18.56)
Urinary catheter	1.54 (0.00–4.32)	1.85 (0.00–3.02)	1.75 (0.39–5.14)	0.94 (0.43–2.24)
Treatment-related prescriptions	12.48 (3.92–19.92)	9.47 (6.64–13.65)	11.06 (5.60–15.92)	9.45 (5.32–14.77)
Total	67.94 (63.34–71.60)	68.80 (65.83–77.64)	73.80 (69.77–75.85)	68.52 (62.70–73.84)
**Fixation rate**,** fixations/min**				
Face	6.10 (4.36–8.73)	7.48 (4.67–12.10)	8.08 (4.15–11.24)	5.76 (3.94–8.06)
Upper limbs	7.00 (1.91–8.71)	4.22 (3.35–6.76)	6.20 (4.45–12.57)	5.78 (3.57–7.36)
Lower limbs	2.38 (0.54–5.08)	4.15 (2.02–6.87)	4.51 (2.37–6.00)	3.70 (2.26–7.93)
Chest/abdomen	10.28 (4.18–15.19)	15.41 (8.99–19.26)	14.82 (10.36–16.90)	13.01 (6.10–18.49)
Insertion sites	0.72 (0.00–2.36)	3.18 (1.86–4.33)	1.73 (1.00–2.62)	2.07 (1.67–2.63)
Endotracheal tube	4.38 (2.70–6.29)	3.88 (1.96–5.11)	4.14 (3.68–5.01)	2.72 (2.10–4.03)
Gastric tube	1.61 (1.04–4.49)	1.94 (0.98–2.89)	1.18 (0.86–2.81)	1.01 (0.72–1.77)
Vital signs monitor	6.62 (2.75–10.18)	8.12 (4.43–12.89)	6.98 (4.95–10.32)	6.05 (4.28–9.45)
Mechanical ventilator	3.84 (2.29–16.12)	6.43 (3.59–10.22)	5.87 (3.25–9.72)	5.64 (3.90–9.93)
Physiological measurement devices	1.91 (0.63–3.34)	3.06 (1.20–4.42)	2.07 (1.24–4.76)	4.24 (2.33–6.12)
Infusion lines and pumps	14.27 (8.86–21.40)	22.53 (17.95–30.59)	19.95 (10.41–29.54)	25.71 (16.97–33.34)
Urinary catheter	2.12 (0.00–6.29)	0.74 (0.00–6.13)	1.34 (0.55–7.17)	1.97 (0.52–3.11)
Treatment-related prescriptions	17.38 (5.37–32.89)	12.42 (8.83–22.22)	13.97 (7.67–21.16)	11.81 (9.43–15.99)
Total	94.00 (87.32–101.33)	103.76 (98.03–108.51)	98.51 (90.04–108.89)	94.99 (89.84–115.70)
**Visit rate**,** visits/min**				
Face	2.07 (1.48–2.89)	2.37 (1.70–3.88)	2.64 (1.99–3.45)	2.70 (1.30–3.68)
Upper limbs	1.61 (1.12–2.72)	1.81 (1.39–2.62)	2.11 (1.61–4.08)	2.37 (1.30–3.44)
Lower limbs	0.34 (0.17–0.72)	0.46 (0.30–1.10)	1.10 (0.46–1.56)	1.12 (0.77–1.38)
Chest/abdomen	1.77 (0.89–2.71)	2.27 (1.73–4.20)	2.74 (2.09–3.94)	2.63 (1.87–3.87)
Insertion sites	0.23 (0.00–0.53)	0.56 (0.31–1.02)	0.76 (0.53–0.96)	0.75 (0.55–0.97)
Endotracheal tube	1.37 (0.95–1.61)	1.51 (0.82–2.72)	2.01 (1.61–2.37)	1.63 (1.09–2.32)
Gastric tube	0.41 (0.25–0.96)	0.74 (0.38–1.47)	0.78 (0.34–1.01)	0.40 (0.34–0.83)
Vital signs monitor	1.30 (0.81–1.75)	1.12 (0.82–1.49)	1.50 (1.20–2.09)	1.82 (1.07–2.85)
Mechanical ventilator	1.36 (0.69–2.32)	1.44 (0.92–2.27)	1.87 (0.84–2.78)	2.05 (1.64–2.96)
Physiological measurement devices	0.40 (0.12–0.75)	0.52 (0.28–1.16)	0.72 (0.33–1.06)	1.17 (0.68–1.45)
Infusion lines and pumps	1.79 (1.08–2.45)	2.20 (1.80–3.11)	2.43 (1.72–3.31)	2.64 (1.94–4.29)
Urinary catheter	0.14 (0.00–0.32)	0.22 (0.00–0.55)	0.25 (0.13–0.39)	0.31 (0.20–0.56)
Treatment-related prescriptions	2.52 (0.65–4.18)	3.03 (1.82–4.61)	2.71 (1.66–3.52)	2.67 (0.93–4.19)
Total	20.19 (16.28–22.04)	23.26 (18.95–29.32)	25.27 (22.14–29.74)	28.76 (20.56–31.04)

Values are presented as median (interquartile range [IQR]; 25th–75th percentile). Fixation duration is expressed as the percentage of the analysis interval spent fixating within each AOI. Fixation and visit rates were standardized to the analysis interval and are expressed as fixations per minute and visits per minute, respectively. Total values represent the sum across the 13 AOIs. AOI, area of interest

### Primary analyses of gaze behavior

Primary GEE analyses were conducted for all 13 AOIs and the total measure for fixation duration, fixation rate, and visit rate (Table 3). After Benjamini–Hochberg correction across the 42 omnibus tests, significant differences among experience groups remained for five fixation-duration measures, four fixation-rate measures, and six visit-rate measures.

**Table 3:** Generalized estimating equation analyses of gaze metrics by emergency and critical care nursing experience group

Gaze metric / AOI	< 1 year Mean (SE)	1–<4 years Mean (SE)	4–<9 years Mean (SE)	≥ 9 years Mean (SE)	Wald χ²	*p* value	FDR q value	Significant Bonferroni-adjusted pairwise comparisons
**Fixation duration, % of analysis interval**								
Face	6.43 (0.93)	7.27 (0.82)	7.60 (0.85)	5.78 (0.43)	5.06	0.168	0.281	
Upper limbs	3.93 (0.43)	2.35 (0.28)	5.48 (1.09)	3.55 (0.18)	15.79	0.001	0.010	< 1 year > 1–<4 years, *p* = 0.023 1–<4 years < 4–<9 years, *p* = 0.049 1–<4 years < ≥ 9 years, *p* = 0.002
Lower limbs	2.54 (0.70)	3.37 (0.94)	2.83 (0.43)	2.95 (0.87)	1.33	0.721	0.818	
Chest/abdomen	7.23 (1.56)	8.93 (1.34)	9.88 (0.73)	8.49 (1.09)	2.62	0.454	0.632	
Insertion sites	1.17 (0.21)	2.54 (0.24)	2.30 (0.53)	2.16 (0.24)	19.21	< 0.001	0.005	< 1 year < 1–<4 years, *p* < 0.001 < 1 year < ≥ 9 years, *p* = 0.008
Endotracheal tube	3.59 (0.52)	2.95 (0.43)	3.74 (0.49)	2.99 (0.22)	3.22	0.359	0.519	
Gastric tube	3.09 (0.66)	2.49 (0.78)	1.86 (0.53)	1.11 (0.33)	10.66	0.014	0.038	< 1 year > ≥ 9 years, *p* = 0.033
Vital signs monitor	4.86 (0.84)	5.22 (0.77)	5.56 (0.56)	6.24 (1.13)	1.12	0.772	0.854	
Mechanical ventilator	5.89 (1.66)	5.96 (1.47)	4.78 (1.24)	5.33 (0.83)	0.38	0.945	0.968	
Physiological measurement devices	1.51 (0.34)	2.22 (0.36)	2.02 (0.44)	3.81 (0.58)	15.31	0.002	0.011	< 1 year < ≥ 9 years, *p* = 0.001
Infusion lines and pumps	9.82 (1.22)	14.64 (1.66)	12.86 (2.06)	14.32 (1.12)	8.46	0.037	0.087	
Urinary catheter	3.03 (0.41)	1.88 (0.52)	2.58 (0.69)	2.06 (0.50)	7.06	0.070	0.140	
Treatment-related prescriptions	12.14 (2.32)	10.94 (1.04)	10.98 (0.97)	10.22 (1.91)	0.40	0.940	0.968	
Total	66.64 (1.47)	69.56 (1.21)	72.68 (1.98)	68.29 (2.04)	11.26	0.010	0.034	—
**Fixation rate**,** fixations/min**								
Face	7.16 (0.90)	8.85 (1.14)	8.70 (1.81)	7.04 (1.02)	1.82	0.610	0.776	
Upper limbs	6.17 (0.43)	4.79 (0.55)	7.83 (0.89)	5.29 (0.32)	14.58	0.002	0.012	1–<4 years < 4–<9 years, *p* = 0.015
Lower limbs	3.96 (1.03)	5.53 (1.34)	4.49 (0.66)	5.24 (1.04)	1.36	0.715	0.818	
Chest/abdomen	11.06 (2.32)	14.72 (1.97)	13.95 (0.98)	12.56 (1.18)	1.67	0.643	0.789	
Insertion sites	1.26 (0.29)	2.94 (0.31)	1.92 (0.34)	2.06 (0.13)	9.83	0.020	0.053	
Endotracheal tube	4.47 (0.40)	4.12 (0.51)	4.60 (0.34)	3.87 (0.23)	7.28	0.064	0.134	
Gastric tube	2.97 (0.59)	2.26 (0.52)	1.71 (0.31)	1.28 (0.44)	12.56	0.006	0.027	—
Vital signs monitor	6.62 (1.05)	8.31 (1.12)	7.49 (0.78)	8.52 (1.52)	1.61	0.658	0.789	
Mechanical ventilator	8.74 (2.15)	8.94 (2.11)	6.61 (1.24)	7.68 (1.54)	0.97	0.809	0.871	
Physiological measurement devices	2.16 (0.48)	2.96 (0.48)	2.74 (0.46)	6.09 (1.68)	11.25	0.010	0.034	—
Infusion lines and pumps	15.27 (2.15)	24.44 (2.64)	20.84 (3.24)	24.41 (2.62)	8.30	0.040	0.089	
Urinary catheter	4.46 (0.64)	2.81 (0.79)	3.37 (0.75)	2.07 (0.57)	10.99	0.012	0.035	< 1 year > ≥ 9 years, *p* = 0.012
Treatment-related prescriptions	19.97 (4.44)	15.15 (1.85)	13.58 (1.18)	12.72 (1.75)	4.23	0.237	0.369	
Total	95.95 (3.91)	104.60 (2.14)	98.30 (2.80)	98.77 (3.18)	4.94	0.176	0.285	
**Visit rate**,** visits/min**								
Face	2.57 (0.53)	2.63 (0.40)	2.74 (0.28)	2.64 (0.48)	0.15	0.986	0.986	
Upper limbs	1.82 (0.22)	2.20 (0.19)	2.70 (0.35)	2.51 (0.35)	8.91	0.030	0.075	
Lower limbs	0.48 (0.09)	0.83 (0.21)	1.05 (0.13)	1.21 (0.08)	24.31	< 0.001	0.001	< 1 year < 4–<9 years, *p* = 0.003 < 1 year < ≥ 9 years, *p* < 0.001
Chest/abdomen	2.16 (0.42)	2.88 (0.30)	3.23 (0.30)	3.00 (0.39)	5.14	0.162	0.281	
Insertion sites	0.33 (0.08)	0.80 (0.11)	0.84 (0.12)	0.87 (0.09)	18.09	< 0.001	0.006	< 1 year < 1–<4 years, *p* < 0.001 < 1 year < 4–<9 years, *p* = 0.001 < 1 year < ≥ 9 years, *p* < 0.001
Endotracheal tube	1.47 (0.17)	1.64 (0.28)	1.89 (0.13)	1.68 (0.17)	6.39	0.094	0.179	
Gastric tube	0.72 (0.14)	0.88 (0.19)	0.77 (0.13)	0.58 (0.16)	2.02	0.567	0.744	
Vital signs monitor	1.26 (0.15)	1.41 (0.16)	1.65 (0.22)	2.00 (0.33)	6.30	0.098	0.179	
Mechanical ventilator	1.78 (0.21)	1.90 (0.24)	1.88 (0.13)	2.25 (0.23)	3.43	0.330	0.496	
Physiological measurement devices	0.46 (0.11)	0.83 (0.16)	0.91 (0.22)	1.08 (0.13)	11.91	0.008	0.029	< 1 year < ≥ 9 years, *p* = 0.001
Infusion lines and pumps	1.76 (0.18)	2.46 (0.28)	2.57 (0.31)	3.01 (0.23)	14.57	0.002	0.012	< 1 year < ≥ 9 years, *p* = 0.001
Urinary catheter	0.21 (0.02)	0.31 (0.07)	0.27 (0.04)	0.31 (0.04)	16.40	< 0.001	0.010	< 1 year < ≥ 9 years, *p* = 0.012
Treatment-related prescriptions	2.68 (0.48)	3.15 (0.42)	2.58 (0.16)	2.77 (0.44)	2.55	0.466	0.632	
Total	19.92 (1.46)	24.25 (1.80)	26.00 (1.54)	27.11 (2.17)	12.07	0.007	0.029	< 1 year < 4–<9 years, *p* = 0.006

Values are estimated marginal means (SE) from generalized estimating equation models. Fixation duration is expressed as the percentage of the analysis interval, whereas estimated marginal means for fixation count and visit count are expressed as rates per minute. Patient ID was specified as the clustering variable, with an exchangeable working correlation structure and robust standard errors. Fixation duration models used a normal distribution with an identity link; fixation and visit count models used negative binomial distributions with log links and the logarithm of analysis interval duration in minutes as an offset. Wald χ² statistics represent Type III tests for experience group. False discovery rate (FDR) *q* values were calculated across all 42 primary omnibus tests (13 AOIs plus the total measure for each of the three gaze metrics) using the Benjamini–Hochberg procedure. Pairwise comparisons were Bonferroni-adjusted and are shown only for outcomes that remained significant after FDR correction. AOI, area of interest; GEE, generalized estimating equation; SE, standard error

Significant differences among experience groups in fixation duration were observed for the upper limbs, insertion sites, gastric tube, physiological measurement devices, and the total measure (FDR *q* < 0.05). For fixation rate, significant differences among experience groups remained for the upper limbs, gastric tube, physiological measurement devices, and urinary catheter. The directions of significant pairwise differences in fixation duration and fixation rate varied across AOIs.

For visit rate, significant differences among experience groups remained for the lower limbs, insertion sites, physiological measurement devices, infusion lines and pumps, urinary catheter, and the total measure. In contrast to fixation duration and fixation rate, all significant pairwise comparisons in visit rate involving the < 1-year group were in the same direction, with lower visit rates in the < 1-year group than in the more-experienced comparison group. For total visit rate, the omnibus test for experience group was significant (Wald χ² = 12.07, *p* = 0.007, FDR *q* = 0.029), and the < 1-year group had a lower rate than the group with 4–<9 years of experience after Bonferroni adjustment (*p* = 0.006). Detailed pairwise comparisons are presented in Table 3.

### Sensitivity analyses

Sensitivity analyses of total visit rate are summarized in Supplementary Tables S1 and S2 (Additional files 1 and 2). After adjustment for patient age, patient sex, and ICU length-of-stay category, the categorical association with experience group remained significant (Wald χ² = 14.69, df = 3, *p* = 0.002). A negative binomial GLMM with a patient-level random intercept also showed a significant association with experience group (*F* = 4.11, *p* = 0.010).

When emergency and critical care nursing experience was modeled as a numeric variable, greater reported experience was associated with a higher total visit rate in the unadjusted GEE model (*B* = 0.020, SE = 0.008, *p* = 0.014) and in the GLMM (*B* = 0.021, SE = 0.008, *p* = 0.009). After adjustment for patient age, patient sex, and ICU length of stay, the association was attenuated and did not reach the conventional level of statistical significance (*B* = 0.018, SE = 0.010, *p* = 0.055).

## Discussion

### Principal findings

A major finding of this study was that during routine post-handover bedside observation, nurses with < 1 year of emergency and critical care nursing experience showed fewer gaze entries per unit time into several patient-, device-, and treatment-related bedside areas than nurses in at least one more-experienced group. Although experience-related differences were also observed in fixation duration and fixation rate, their directions varied across AOIs. In contrast, all significant pairwise differences in visit rate involving the < 1-year group were in the same direction. Thus, the clearest experience-related difference was not a uniform increase or decrease in how long or how often nurses fixated on individual areas, but in how frequently their gaze entered multiple bedside areas during the initial observation.

This finding extends previous critical care eye-tracking research by showing that experience-related differences can be identified during naturally occurring observation of actual critically ill patients, without predefined abnormalities, a prescribed observation sequence, or a fixed observation duration. In this open-ended clinical situation, differences associated with experience were most directionally consistent in visit rate across multiple potential sources of patient information rather than in the amount of fixation on individual areas.

### Task-dependent differences in gaze behavior

The meaning of gaze behavior is likely to depend on the clinical task in which it occurs. Research conducted during extended routine ICU care has primarily described how visual attention is distributed among patients, monitoring systems, ventilators, and other work-related targets [18]. Experience-related comparisons have also been conducted during a more constrained, device-specific task in which the relevant visual targets were largely determined by the ventilator interface [19]. Simulated shift-start safety checks have examined gaze behavior during detection of predefined errors; in the overall analysis of one such study, more revisits to predefined AOIs were associated with a greater likelihood of missing errors [20]. More recent simulation research has also shown that time pressure can alter gaze behavior and reduce error detection during an ICU handover safety check [21].

Post-handover bedside observation has a different task structure. Nurses do not know which bedside information sources will contain clinically relevant findings in advance and must observe the patient, invasive devices, monitoring equipment, and treatment-related information in the context of the evolving clinical situation. Although the revisit measure used in previous simulated safety-check research is not identical to the visit-rate measure used here, the contrast suggests that repeated gaze entries may have different meanings across tasks. During predefined error detection, repeated revisits may reflect difficulty locating a target, whereas during open-ended bedside observation, repeated gaze entries into bedside areas may reflect repeated checking of potential information sources. This task-dependent interpretation may help explain why visit rate showed a more directionally consistent relationship with experience than fixation duration or fixation rate in this study.

### Experience and the early process of clinical judgment

The observed experience-related pattern may reflect differences in how individual bedside areas are approached as sources of clinical information. Nurses with limited experience may attend to some areas as discrete elements to be checked, whereas with greater experience the same areas may be approached as contextually relevant sources of patient information. This interpretation remains hypothetical because this study did not assess why nurses looked at each area or how the information obtained was interpreted.

The NCJMM provides a framework for considering the clinical relevance of this pattern. Recognize Cues concerns identifying relevant and important information, followed by Analyze Cues, in which findings are related and interpreted [13, 14]. Gaze entry does not establish that information was recognized or interpreted as a clinically relevant cue, but it provides an opportunity for visually available information to be encountered. The lower visit rates observed in the < 1-year group may therefore indicate fewer opportunities to encounter potential cues, rather than poorer cue recognition itself. Such differences may relate to the range of information available for subsequent patient assessment and prioritization and may have potential relevance to patient safety. Tanner’s clinical judgment model similarly emphasizes that noticing is shaped by previous experience, knowledge of the patient, expectations, and clinical context [12]. However, eye-tracking data alone cannot determine whether cues were recognized, integrated, or acted upon appropriately.

### Educational implications

The findings suggest potential applications for critical care nursing education, including orientation of nurses entering critical care practice and simulation-based debriefing. Rather than showing nurses with limited experience where more-experienced nurses look, eye-tracking feedback could prompt reflection on why particular patient-, device-, or treatment-related areas are observed, what information is sought, and how observations relate to other potential cues. Gaze recordings could therefore support discussion of observation strategies and clinical reasoning without encouraging simple imitation of another nurse’s gaze pattern. Such use would be most appropriate as a reflective or formative educational tool, not as a standalone measure of competence. However, this study did not evaluate an educational intervention, and whether gaze-based debriefing improves cue recognition, clinical judgment, or patient assessment remains to be tested.

### Limitations

This study has several limitations. First, it was conducted at a single institution, and the 66 observation sessions involved only 18 unique patients. However, patient-level clustering was accounted for using GEE with robust covariance estimation, and the central finding for total visit rate was also examined using a patient-level random-intercept GLMM. Nevertheless, inference from GEE may be less reliable when the number of independent clusters is small [27], and the limited number of patient clusters may have reduced the precision of the estimates. The consistency of the central finding across the GEE and GLMM analyses supports its robustness but does not eliminate uncertainty related to the small number of clusters. In addition, wearing mobile eye-tracking glasses and awareness that their gaze was being recorded may have influenced their usual observation behavior.

Second, the primary analyses involved 42 omnibus comparisons across 13 AOIs and the total measure for three gaze metrics. The Benjamini–Hochberg procedure was applied across all 42 tests to control the false discovery rate, and Bonferroni adjustment was used for subsequent pairwise comparisons. Nevertheless, given the number and related nature of the gaze outcomes, individual AOI-specific findings should be interpreted cautiously, with greater emphasis placed on consistent patterns across related measures rather than on isolated statistically significant results.

Third, gaze behavior may have been influenced by patient, nurse, and situational factors in addition to nursing experience. Nurse age also differed across experience groups and was not included in the sensitivity models; therefore, age-related and experience-related influences could not be disentangled in this study. The association between experience group and total visit rate remained significant after adjustment for patient age, patient sex, and ICU length of stay; however, other factors—including illness severity, treatment status (such as vasopressor use), patient familiarity, prior technology exposure, educational background, individual cognitive strategies, and workload—were not fully captured or modeled and may have contributed to the observed patterns. When experience was modeled numerically, the association with total visit rate was attenuated after covariate adjustment and did not reach the conventional level of statistical significance. Therefore, the findings do not establish a simple linear or dose–response relationship between years of experience and gaze behavior, and residual confounding cannot be excluded.

Fourth, years of emergency and critical care nursing experience were used as an operational grouping variable and should not be interpreted as validated levels of expertise, competence, or proficiency in bedside observation. Although the study protocol specified a target of 16 participants per group based on a calculation using G*Power 3 [24], this target was not achieved in all final experience groups. Pairwise estimates, particularly those involving the smallest group, may therefore be imprecise, and nonsignificant differences should not be interpreted as evidence of equivalence.

Fifth, eye tracking identifies where gaze is directed but cannot establish whether information was consciously perceived, recognized as clinically relevant, interpreted, integrated with other findings, or acted upon. Accordingly, gaze behavior should not be regarded as a direct measure of clinical competence, observation quality, or clinical judgment. AOI development and gaze-to-AOI mapping involved researcher judgment, and formal inter-rater reliability was not quantified, although all recordings were reviewed by a three-member analysis team and ambiguous or discrepant judgments were resolved by consensus. The analyses also did not examine gaze sequences, transitions between AOIs, or scan paths; therefore, they cannot determine whether the observed visit-rate differences reflected sequential or locally focused viewing, repeated returns to information sources, or switching among AOIs. Future studies combining eye tracking with transition analyses, verbal reports, observation accuracy, and subsequent clinical reasoning or actions are needed to examine these processes directly.

## Conclusions

During routine post-handover bedside observation of critically ill patients, gaze behavior differed according to nurses’ emergency and critical care nursing experience. Although experience-related differences were identified across fixation duration, fixation rate, and visit rate, visit rate showed the most directionally consistent pattern: nurses with < 1 year of experience showed fewer gaze entries per unit time into several patient-, device-, and treatment-related areas than nurses in at least one more-experienced group. The central categorical experience-group finding for total visit rate remained significant in the covariate-adjusted GEE and in the GLMM sensitivity analysis.

The findings suggest that experience-related differences in bedside observation may involve not only how much visual attention is directed to individual areas, but also the frequency with which nurses direct gaze toward multiple potential sources of patient information. This may be relevant to the early process through which potential clinical cues become visually available for subsequent recognition and analysis. However, gaze behavior alone cannot determine whether cues were recognized, interpreted, integrated, or acted upon and should not be regarded as a measure of clinical competence or observation quality. Further research combining eye tracking with gaze-transition analyses and measures of cue recognition and clinical reasoning is needed to clarify how visual observation develops in relation to clinical judgment.

## Supplementary Information

Below is the link to the electronic supplementary material.

Supplementary Material 1: Additional file 1: Supplementary Table S1. Robustness analyses of total visit rate using categorical experience groups.

Supplementary Material 2: Additional file 2: Supplementary Table S2. Sensitivity analyses of total visit rate using self-reported year of emergency and critical care nursing experience as a numeric predictor.

## Data Availability

The datasets generated and/or analyzed during the current study are not publicly available because of ethical considerations and restrictions related to patient confidentiality. De-identified data may be available from the corresponding author on reasonable request and with appropriate ethical approval.

## Abbreviations

AOI: Area of interest
FDR: False discovery rate
GEE: Generalized estimating equation
GLMM: Generalized linear mixed model
ICU: Intensive care unit
IQR: Interquartile range
I-VT: Velocity-Threshold Identification
NCJMM: National Council of State Boards of Nursing Clinical Judgment Measurement Model
SD: Standard deviation
SE: Standard error
STROBE: Strengthening the Reporting of Observational Studies in Epidemiology
