# Supplementary material for: Visual attention during post-handover bedside observation of critically ill patients: an eye-tracking comparison across emergency and critical care nursing experience groups
Source: BMC Nurs. 2026 Sep 2;25:832. doi: 10.1186/s12912-026-05324-1 (PMC13563798; doi:10.1186/s12912-026-05324-1)
Supplement: Supplementary file 1 [file 12912_2026_5324_MOESM1_ESM.docx]

**Supplementary Table S1. Robustness analyses of total visit rate using categorical experience groups**

| **Sensitivity analysis** | **Adjustment** | **Test statistic** | ***p* value** |
| --- | --- | --- | --- |
| Primary negative binomial GEE | None | Wald χ² (3) = 12.07 | 0.007 |
| Covariate-adjusted negative binomial GEE | Patient age, patient sex, and ICU length of stay | Wald χ² (3) = 14.69 | 0.002 |
| Negative binomial GLMM | None | *F* (3, 62) = 4.11 | 0.010 |

Note. All analyses used total visit count as the outcome, a negative binomial distribution with a log link, and the logarithm of analysis interval duration in minutes as an offset. GEE models specified patient ID as the clustering variable with an exchangeable working correlation structure; robust standard errors were used. The GLMM included a patient-level random intercept. The covariate-adjusted GEE included patient age, patient sex, and ICU length-of-stay category. These analyses assess robustness of the same central outcome and were not treated as a separate family of primary hypotheses. GEE, generalized estimating equation; GLMM, generalized linear mixed model; ICU, intensive care unit.
