# Supplementary material for: Visual attention during post-handover bedside observation of critically ill patients: an eye-tracking comparison across emergency and critical care nursing experience groups
Source: BMC Nurs. 2026 Sep 2;25:832. doi: 10.1186/s12912-026-05324-1 (PMC13563798; doi:10.1186/s12912-026-05324-1)
Supplement: Supplementary file 2 [file 12912_2026_5324_MOESM2_ESM.docx]

**Supplementary Table S2. Sensitivity analyses of total visit rate using self-reported year of emergency and critical care nursing experience as a numeric predictor**

| **Sensitivity analysis** | **Adjustment** | ***B*** | **SE** | ***p* value** |
| --- | --- | --- | --- | --- |
| Negative binomial GEE | None | 0.020 | 0.008 | 0.014 |
| Negative binomial GLMM | None | 0.021 | 0.008 | 0.009 |
| Covariate-adjusted negative binomial GEE | Patient age, patient sex, and ICU length of stay | 0.018 | 0.010 | 0.055 |

Note. All analyses used total visit count as the outcome, a negative binomial distribution with a log link, and the logarithm of analysis interval duration in minutes as an offset. GEE models specified patient ID as the clustering variable with an exchangeable working correlation structure and robust standard errors; the GLMM included a patient-level random intercept. The covariate-adjusted GEE included patient age, patient sex, and ICU length-of-stay category. Participants reported their current year of emergency and critical care nursing experience (e.g., first year, second year) on the questionnaire; exact elapsed experience duration in months was not collected. *B* represents the change in the log visit rate associated with a one-unit increase in this self-reported year-of-experience variable. Accordingly, this analysis was treated as a numeric sensitivity analysis rather than an analysis of exact continuous experience duration. GEE, generalized estimating equation; GLMM, generalized linear mixed model; ICU, intensive care unit; SE, standard error.
